# A286 THE ROLE OF A GUT HORMONE VERSUS NUCLEAR FACTOR-KAPPA B IN PATIENTS WITH NON-ALCOHOLIC FATTY LIVER DISEASE

**DOI:** 10.1093/jcag/gwac036.286

**Published:** 2023-03-07

**Authors:** M Abdel-Samiee, E Abdelsameea, A Badran

**Affiliations:** 1 National Liver Institute, Menoufia University, Menoufia; 2 Specialized Medical Center-Damas Liver Center, Mansora, Egypt

## Abstract

NOT PUBLISHED AT AUTHOR’S REQUEST

**Please acknowledge all funding agencies by checking the applicable boxes below:**

None

**Disclosure of Interest:**

None Declared

